# Missed diagnosis of Fabry disease: should we screen patients with multiple sclerosis?

**DOI:** 10.1007/s10072-023-06962-y

**Published:** 2023-07-22

**Authors:** Petra Rekova, Ivana Kovarova, Tomas Uher, Barbora Srpova, Gabriela Dostalova, Ales Linhart, Manuela Vaneckova, Dominika Stastna

**Affiliations:** 1 Department of Neurology and Centre of Clinical Neuroscience, First Faculty of Medicine, Charles University in Prague and General University Hospital, Prague, Czechia; 2 Second Department of Internal Cardiovascular Medicine, First Faculty of Medicine, Charles University in Prague and General University Hospital, Prague, Czechia; 3 Department of Radiology, First Faculty of Medicine, Charles University in Prague and General University Hospital, Prague, Czechia

**Keywords:** Fabry disease, A variant of unknown significance, Multiple sclerosis, Misdiagnosis, Screening, Differential diagnosis

## Abstract

**Introduction:**

Fabry disease (FD) can be undiagnosed in the context of multiple sclerosis (MS) due to similar clinical and paraclinical features. Our study aimed to determine the prevalence (and the necessity of screening) of FD among patients with possible or definite MS.

**Methods:**

In this prospective monocentric observational study, we included consecutive patients enrolled between May 2017 and May 2019 after the first clinical event suggestive of MS. All patients underwent FD screening using dried blood spots in a stepwise manner combining genetic and enzyme testing. Patients were followed until May 2022.

**Results:**

We included 160 patients (73.1% female, mean age 33.9 years). The 2017 revised McDonald’s criteria for definite MS were fulfilled by 74 (46.3%) patients at the time of study recruitment and 89 (55.6%) patients after 3–5 years of follow-up. None of the patients had a pathogenic GLA variant, and four (2.5%) had a variant of unknown significance (p.A143T, p.S126G, 2 × p.D313Y). In two of these patients, the intrathecal synthesis of oligoclonal bands was absent, and none had hyperproteinorachia or pleocytosis in cerebrospinal fluid. Detailed examination of FD organ manifestations revealed only discrete ocular and kidney involvement in two patients.

**Conclusion:**

The prevalence of FD in the population of suspected or definite MS patients does not appear to be high. Our results do not support routine FD screening in all patients with a possible diagnosis of MS, but there is an urgent need to search for red flags and include FD in the differential diagnosis of MS.

## Introduction

Multiple sclerosis (MS) is an autoimmune demyelinating disorder of the central nervous system (CNS) with a broad spectrum of clinical symptoms. Both the clinical features of the disease and laboratory investigations, such as magnetic resonance imaging (MRI) and cerebrospinal fluid (CSF) analysis, are used in the diagnosis [1]. The main aim is to establish the dissemination in space and time of the MS-typical clinical and imaging presentation caused by the lesions in the CNS and to rule out other diseases that might mimic MS. Due to the high variability of symptoms originating from many areas of the CNS and the absence of specific biomarkers confirming the diagnosis, the differential diagnosis of MS can be difficult [2]. As much as 5–10% of patients with MS are misdiagnosed due to misinterpretation and misapplication of MS clinical and radiographic diagnostic criteria with overreliance on MRI abnormalities and non-specific neurological symptoms [3, 4].

One of the recognised but sometimes underestimated mimics of MS is Fabry disease (FD). Several studies have described patients with FD who were initially misdiagnosed with MS [5–7] or later found to have both conditions [8]. FD is a progressive, X-linked inherited disorder of glycosphingolipid metabolism due to deficient or absent alpha-galactosidase-A (AGAL) activity caused by over 1000 known disease-associated variants in the GLA gene [9]. The AGAL deficiency results in the accumulation of globotriaosylceramide and other glycosphingolipids in various cell types, including renal, cardiac, nerve, and endothelial cells [10]. The estimated incidence of FD is 1 per 40,000–1 per 60,000 [11]. However, some studies indicate a possible higher prevalence [12]. The diagnosis of FD has important therapeutic implications as a specific therapy is available.

FD is a disease with a broad spectrum of heterogeneously progressive clinical phenotypes due to the different residual levels of AGAL activity, with contributions from modifying factors. The classical, and most severe, manifestation of the disease phenotype is in hemizygous males in childhood or adolescence. Symptoms include acroparesthesias due to peripheral neuropathy, angiokeratomas, hypohidrosis, gastrointestinal symptoms, corneal dystrophy (cornea verticillata), and later cardiac, renal, and CNS involvement. On the other end of the spectrum are milder late-onset or “non-classical” phenotypes. These types are mostly observed in males with a higher degree of residual GLA activity or in symptomatic heterozygous females with primarily one organ system’s impairment (e.g., the cardiac, renal, or nervous system), cerebrovascular disease, and the absence of the classical signs of the disease [10–13].

FD can be misdiagnosed as MS or remain undiagnosed in patients with MS for several reasons (Table 1). Both diseases can present clinically in a “relapsing–remitting” manner and with similar symptoms. Sensory complaints (paresthesia in MS; painful, burning acral sensations in FD) are the most common manifestations of both diseases and can easily be confused. These two diseases also coincide in the occurrence of vertigo and non-specific complaints such as fatigue, the progression of neurological disability, and accentuation of difficulties on exertion or in the heat. Additionally, some disseminated white matter MRI lesions in FD may resemble MS lesions and fulfil criteria for dissemination in space [4]. The last major factor mimicking MS is CSF analysis. The typical findings in patients with MS reflect the inflammatory nature of the disease and include mild pleocytosis, mild protein increase, and, usually, the presence of oligoclonal bands (OCBs) [14]. It was discovered that the CSF of patients with FD could also show a picture of aseptic or chronic meningitis with mild to moderate pleocytosis and mild hyperproteinorachia caused by the aseptic inflammatory process [15, 16]. Very rarely, CSF-restrictive OCBs can also be found [7, 17]. In contrast, OCBs are present in approximately 90% of MS patients [4, 18].

**Table 1:** The classic and overlapping findings in multiple sclerosis and Fabry disease

	Multiple sclerosis	Fabry disease	Possible overlapping findings
Clinical findings	A clinical attack lasts at least 24 h in the absence of fever or infection; typically not sudden onset	Sudden onset of symptoms in case of cerebrovascular event	Relapsing–remitting manner of complaints, and the progression of neurological disability in time
	Commonly relapsing–remitting manner of complaints; the progression of neurological disability in time	The progression of neurological disability in time	
	A common first presentation is unilateral optic neuritis	Cornea verticillata	
	Sensory signs (reduced fine touch, vibration sense, joint position sense, tight band-like sensation around the trunk, neuropathic pain), Lhermitte’s phenomenon	Burning acroparesthesias (peripheral small fibre neuropathy)	Sensory symptoms
	An upper motor neuron lesion with increased tone or spasticity, pyramidal weakness, hyperreflexia, extensor plantar responses	Cardiac, renal, gastrointestinal involvement, angiokeratomas, hypohidrosis	
	Brainstem syndromes (diplopia, oscillopsia, vertigo, isolated sixth nerve palsy, gaze-evoked nystagmus, internuclear ophthalmoplegia)	Vertigo, tinnitus, hearing loss	Vertigo, tinnitus, hearing loss
	Fatigue, difficulties on exertion or in the heat	Fatigue, difficulties on exertion or in the heat	Fatigue, difficulties on exertion or in the heat
MRI	At least one T2 hypersignal lesion in two locations t of 4 typical areas of the CNS (juxtacortical/intracortical, periventricular, infratentorial, spinal cord); inflammatory aetiology	Findings consistent with affected vascular territory due to increased prevalence of cerebrovascular events; can occur cerebral micro-bleedings and basilar artery elongation and dilatation	Subcortical and periventricular white matter hyperintensities
	Demyelinating/inflammatory lesions	Ischemic lesions	
	Typical perivenular distribution, ovoid shape, perpendicular to the ventricles (Dawson fingers) Ovoid lesions in corpus callosum	T2 hyperintense white matter lesions (subcortical, periventricular, relative sparing of midline and infratentorial structures), typically no corpus callosum	
	Brainstem and spinal cord lesions are typically small and usually peripherally located	Infratentorial lesions are ischemic, thus usually centrally located	
	Gadolinium enhancement of the new lesion for about a month	No gadolinium enhancement	
	Spinal cord lesions are common	No spinal cord involvement	
CSF	OCBs in most of the patients, may be mild pleocytosis (typically lymphocytic), an increase in immunoglobulin concentrations	The characteristic pattern is not known	Pleocytosis, mild hyperproteinorachia, rarely OCBs

*CNS*, central nervous system; *MRI*, magnetic resonance imaging; *FD*, Fabry disease; *CSF*, cerebrospinal fluid; *OCBs*, oligoclonal bands; based on [1, 4, 15, 16, 18–22]

Our study aimed to determine the prevalence of FD among patients with possible or definite MS diagnoses and to evaluate the necessity of including genetic and enzyme screening in the standard testing protocol for differential diagnosis of all suspected MS patients.

## Materials and methods

### Population of the study

In this prospective monocentric study, we included patients admitted between May 2017 and May 2019, after their first clinical event suggestive of MS, to the largest Czech MS centre. Patients who remained in the MS centre for follow-up until May 2022 were included in the final analysis.

### Data collection

Healthcare professionals collected clinical and laboratory data (Table 2) as part of the routine examination process and patient follow-up. FD was diagnosed using dried blood spots via CentoCard (CentoGene AG) in a stepwise manner combining genetic and enzyme testing as described previously [13]. Shortly, in males: enzymatic activity, globotriaosylsphingosine (lyso-Gb3) quantification, if positive, followed by GLA gene sequencing; and in females: GLA sequencing followed by lyso-Gb3. Patients with positive screening results were referred to a specialised FD centre for further clinical and laboratory investigations and management, including family screening. All examinations in the FD centre were performed according to the internal protocol of the FD centre for initial disease evaluation as described in a previous publication [23].

**Table 2:** Demographic, clinical, and laboratory characteristics

	All	Definite MS as of May 2022
Study population	160	89
Age in years, mean (SD)	33.94 (8.24)	33.94 (8.54)
Female (%)	117 (73.13%)	65 (73.03%)
History of sensory symptoms (%)	88 (55.00%)	49 (55.06%)
Initial CSF analysis		
Two or more OCBs (%)	111 (69.38%)	78 (87.64%)
Pleocytosis (%)	56 (35.00%)	35 (39.33%)
Hyperproteinorachia (%)	35 (21.88%)	14 (15.73%)
Pathological GLA variants carriers (%)	0	0
GLA variants of unknown significance or probably benign carriers (%)	4 (2.50%)	2 (2.25%)

*MS*, multiple sclerosis; *CSF*, cerebrospinal fluid; *OCBs*, oligoclonal bands

### Statistical analyses

The characteristics of the population were summarised using means for continuous variables and frequencies (%) for categorical variables. Patients with genetically proven GLA variants were described separately (Table 3). Data analyses were performed by SPSS version 22.0.0.0.

**Table 3:** The genetic, biochemical, and clinical details of patients with proven GLA variants

Patient	Sex	Age at first symptoms	EA plasma (nmol/mL/h)	EA leukocytes (nmol/mg/h)	Lyso-Gb3 DBS (ng/mL)	First neuro symptoms	OCBs
c. 427 G > A, p. Ala143Thr (p.A143T)							
1.0	F	49	4.59	33.3	1.0	Combined sensory-motor	No
1.1	F	NA	1.35	38.8	NA	None	NA
1.2	M	NA	0.80	25.7	NA	None	NA
c. 376A > G, p. Ser126Gly (p.S126G)							
2.0	F	29	6.34	70.4		Sensory	Yes
2.1	F	NA	4.63	46.2	NA	None	NA
c. 937G > T, p. Asp313Tyr (p.D313Y)							
3.0	F	44	2.33	47.6	0.8	Motor and sensory	Yes
4.0	F	27	NA	NA	0.8	Sensory	No

*EA*, enzyme activity; *DBS*, dry blood spot; *OCBs*, two or more oligoclonal bands only in cerebrospinal fluid; *F*, female; *M*, male; *NA*, not applicable/not available; controls: alpha-galactosidase-A in leukocytes 25–103 nmol/mg/h, mean value ± SD 59.7 ± 14.6 nmol/mg/h, *n* = 477, in plasma 2.4–19.4 nmol/mL/h, mean value ± SD 6.1 ± 2.8 nmol/mL/h, *n* = 322

## Results

Baseline clinical and laboratory characteristics are described in Table 2. The 2017 revised McDonald’s criteria for definite MS [1] were fulfilled by 74 out of 160 (46.3%) enrolled patients at the time of study recruitment. After 3–5 years of follow-up, this number increased to 89 (55.6%) patients. When compared to the whole study population, these 89 patients had a higher percentage with two or more OCBs (87.64%) or pleocytosis (39.33%) and a lower percentage with hyperproteinorachia (15.73%) in the initial CSF examination (Table 2). Four out of all 160 (2.50%) patients (all females) had a positive screening result. The genetic, biochemical, and clinical details of positively screened patients are provided in Table 3.

### Neurological symptoms of GLA variant carriers

None of the patients had a recognised pathological variant of the GLA gene. However, four female patients had a variant of the GLA gene considered of unknown significance (p.A143T, p.S126G, 2 × p.D313Y). After a 3–5-year follow-up, only one (p.D313Y) did not fulfil the 2017 revised McDonald’s MS criteria. None of these patients had hyperproteinorachia or pleocytosis in the CSF. Three of the patients with GLA variants experienced sensory symptoms. In a patient with the p.A143T variant (patient 1.0), intrathecal OCB synthesis was absent, and in one patient with p.D313Y (patient 4.0), CSF examination showed only one non-corresponding OCB.

**Fig. 1:**
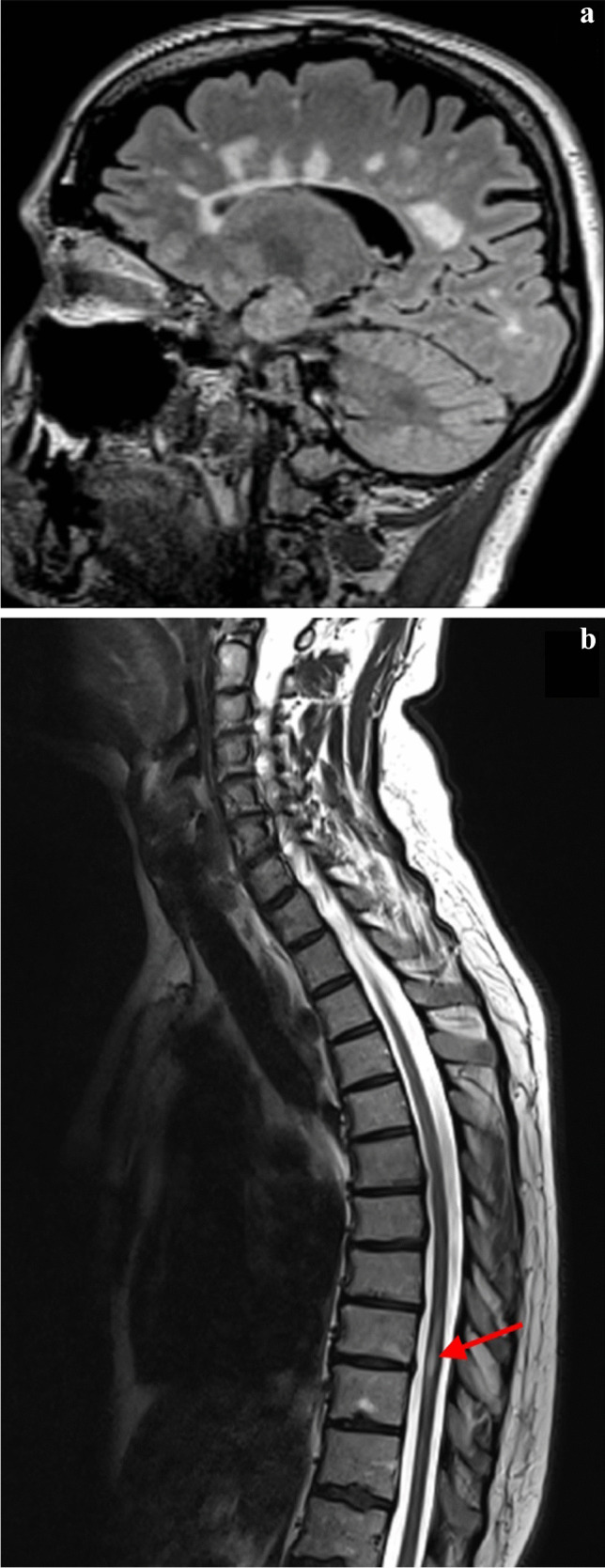
MRI of the patient 1.0. Sagittal FLAIR shows callososeptal hyperintensities radiating from the lateral ventricles with a typical perpendicular orientation (**a**). Sagittal T2 weighted image shows typical intramedullary lesion (**b**)

Initial neurological manifestations of patient 1.0 included sensory and motor symptoms and correlated with the focal spinal cord lesion located in the Th9/10 segment. Brain MRI revealed multiple supra- and infratentorial white matter lesions without contrast enhancement (Fig. 1). A detailed comprehensive examination revealed no other explanatory aetiology besides the GLA gene variant. After a sensitive relapse in August 2020, the clinically isolated syndrome (CIS) diagnosis was reclassified to relapsing–remitting MS.

**Fig. 2:**
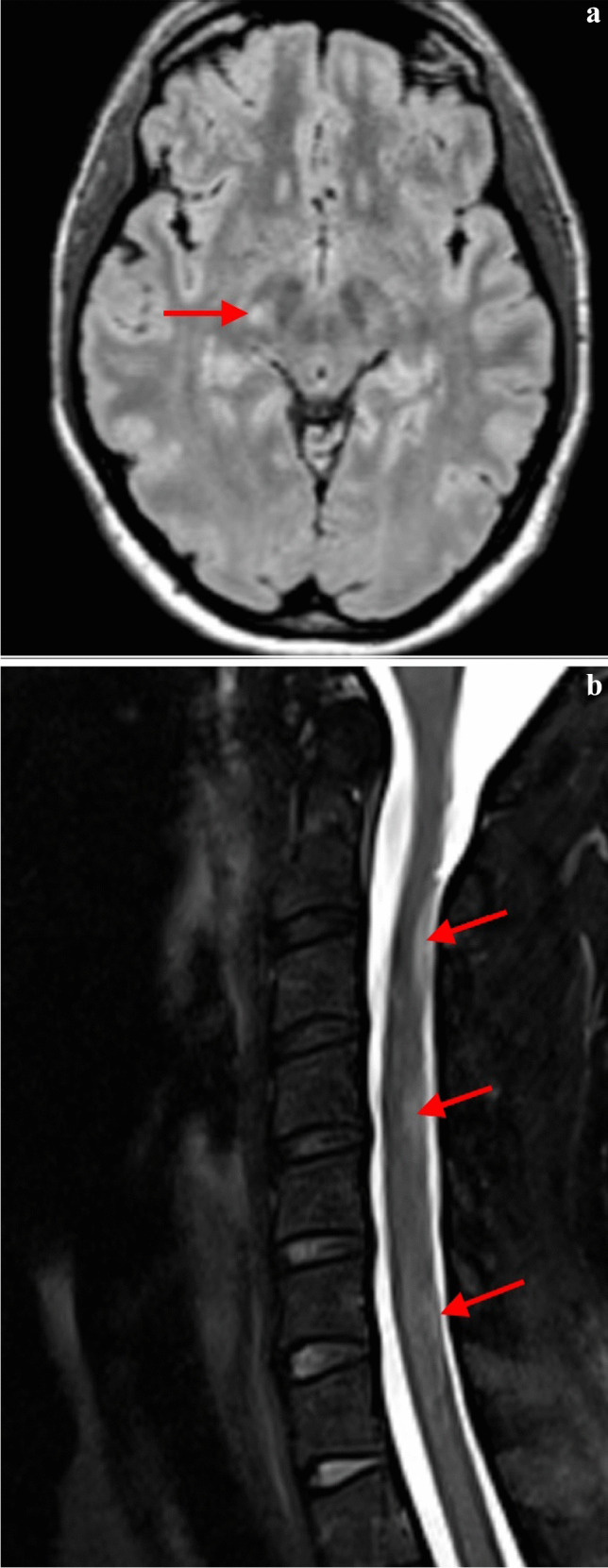
MRI of the patient 2.0. Axial FLAIR shows small lesion (**a**) and multiple typical laterodorsal hypersignal lesions in cervical spinal cord (**b**)

The second patient was diagnosed as a carrier of p.S126G (patient 2.0). Her first symptom was left upper limb hypoesthesia. Brain and spinal cord MRI showed two supratentorial and three intramedullary lesions (Fig. 2). Given the intrathecal OCB synthesis, this patient fulfilled the criteria for definitive MS. Detailed analysis of the organ manifestations of FD showed no symptoms typical of FD or Fabry organ involvement.

**Fig. 3:**
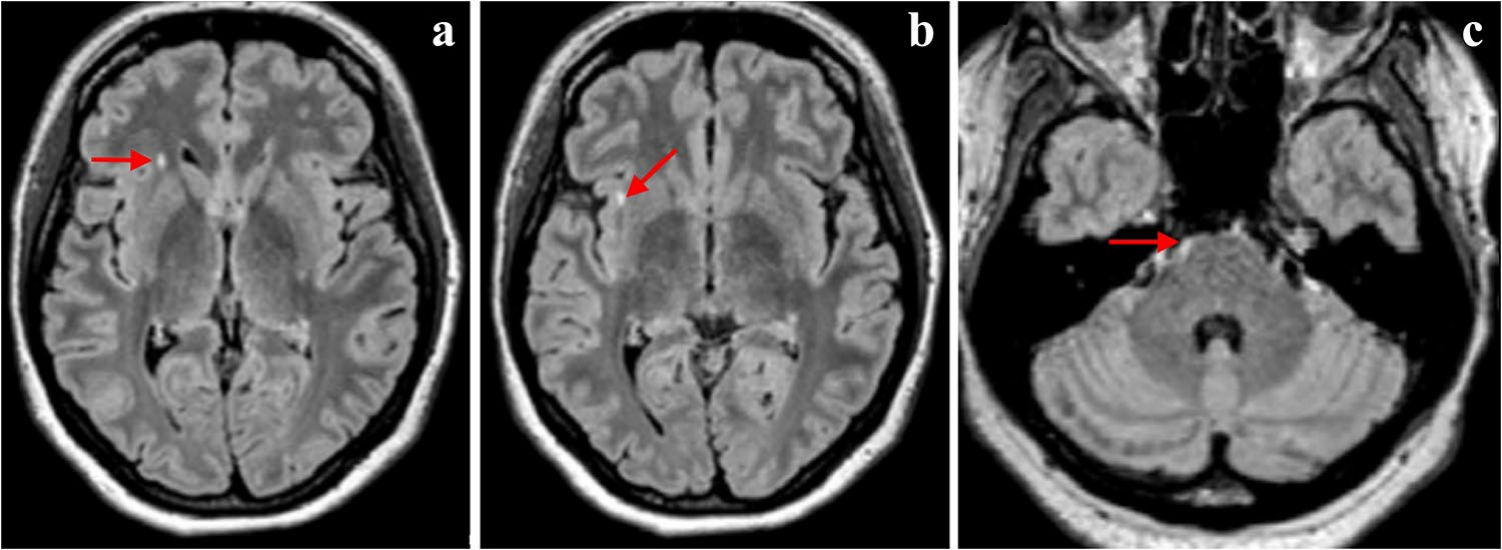
MRI of the patient 3.0. Discrete pathological MRI involvements fulfil MRI criteria with typical lesions distribution for MS. Axial FLAIR show small lesions in the frontal lobe (**a**, **b**), one in the typical subcortical region (**b**) and in the pons (**c**)

**Fig. 4:**
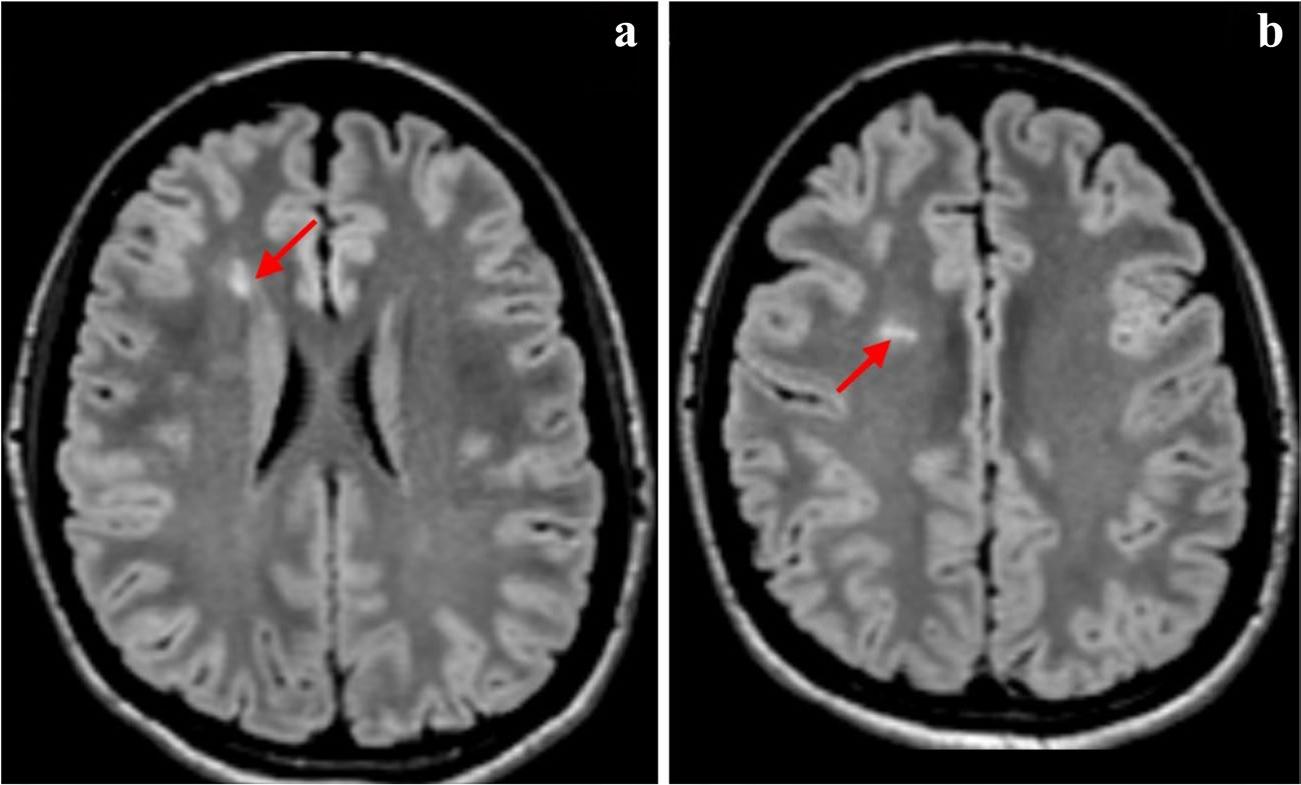
MRI of the patient 4.0. Discrete pathological MRI involvements. Axial FLAIR shows two small lesion in the periventricular white matter (**a**, **b**)

The two remaining patients were shown to carry the p.D313Y variant. The first one (patient 3.0) developed a sudden onset of left-sided hemiparesis and left-sided paraesthesia. Initially, stroke as an underlying course was considered. MRI of the brain and cervical spine revealed one pontine and two supratentorial lesions (Fig. 3). CSF examination showed the presence of 11 CSF-restricted OCBs, and patient 3.0 fulfilled definite MS criteria. The second patient with the p.D313Y variant (patient 4.0) contacted a physician for paraesthesia on the dorsum of the hands in 2017. These complaints resolved spontaneously within weeks and reappeared in 2019. That year, an MRI of the cervical spinal cord and brain showed two hyperintensities in the frontal lobe (Fig. 4). This patient was still diagnosed with CIS at the end of the follow-up.

### FD phenotype examination, family screening

Except for patient 4.0, all patients agreed to a detailed examination by FD specialists in our referral FD centre. After mild, temporary proteinuria, patient 1.0 underwent a renal biopsy with no typical storage vacuoles being found. However, mild interstitial fibrosis and podocytopathy were shown. Moreover, increased endoplasmatic reticulum (ER) stress and signs of unfolded protein response activation were observed and were associated with alfa-GAL misprocessing in this patient. [24]. The offspring of patient 1.0 were also genetically tested. At the time of examination, the son (patient 1.1) was 27 years old, and the daughter (patient 1.2) was 29. The same GLA variant (p.A143T) was found in both offspring. Patient 1.1 also underwent the examination (except for ophthalmological, which he did not attend) at the FD centre. The tests performed did not show any FD organ manifestations. Patient 1.2 refused a detailed organ assessment. Interestingly, the proband’s father died suddenly, apparently of heart disease, at age 49.

In patient 2.0, the detailed examination did not reveal any FD organ manifestation. Family screening of patient 2.0 led to identifying the same variant in the proband’s mother. An examination in the FD centre did not show any organ involvement attributable to FD. In patient 3.0, only discrete ocular signs (tortuous conjunctival vessels and ocular changes suspicious of incipient Fabry cataract) were found. Relatives of both patients with the p.D313Y variant refused the genetic or clinical examination.

## Discussion

Clinical and paraclinical findings in MS may mimic FD and vice versa. It is crucial to differentiate between the two diseases accurately or to recognise the presence of both. Early initiation of treatment before the development of irreversible tissue damage improves the quality of life and prognosis of patients with both diseases. In our 160-patient cohort, we identified four patients and three different types of GLA variants. The atypical findings not supporting MS diagnosis in these four patients included the absence of intrathecal OCB synthesis in two of these patients. Otherwise, the patients did not differ substantially from the rest of the cohort, and there is no reason to doubt the accuracy of their diagnosis of MS. All identified GLA variants are missense ones. The clinical significance of these variants has been the subject of long expert debate. It is worth mentioning that all these variants (p.A143T, p.S126G, p.D313Y) were initially considered disease-causing [25]. Later, their influence on phenotypic manifestations attributable to FD in their carriers was questioned.

The p.A143T is a relatively common variant with an incidence of 1 in every 3800 live births [26]. While some infants with a positive screening for this variant have shown a family history of organ involvement related to FD [12], its pathogenicity remains debated. The carriers of p.A143T do not develop lysosomal storage [27]. The explanation of possible organ manifestations can be the mechanism of AGALopathy, a lysosomal storage-independent pathogenetic factor in FD which was proposed recently [24]. Although the clinical course from a neurological point of view and findings on brain and spinal cord MRI support the definite diagnosis of MS in patient 1.0, the absence of OCBs in the CSF analysis, together with mild renal involvement, does not exclude the possibility that the presence of a GLA variant might potentiate neurological symptoms associated with MS. Furthermore, the enzyme analysis of the patient’s son showed an attenuated biochemical phenotype with low AGAL activity in plasma and borderline activity in leukocytes. Despite normal clinical findings, it might indicate the influence of the variant, at least at the biochemical level. Additionally, intracellular misprocessing of AGAL in p.A143T has been described [24]. However, interpretation of the effect of the variant on the clinical phenotype in the context of its interaction with other genetic, environmental, or even X chromosome inactivation factors is difficult and remains a challenge for further investigation.

Based on the enzyme activity analysis, lyso-GB3 values, and clinical symptoms in a large group of GLA gene variant carriers, the p.S126G variant (frequency 0.07% in non-Finnish Europeans [28]) was not found to be disease-causing and is currently considered a VUS [29]. Our patient 2.0 with variant p.S126G had CSF and MRI findings typical for MS, which also correlated with typical clinical sensory symptoms. Moreover, MS as the cause of the patient’s difficulties was supported by the course of the disease and the normal results of other organ examinations. Neither the patient nor her mother had any typical manifestation that could be attributed to FD.

The p.D313Y variant is the most common of the three variants we found in the population (frequency 0.44% in non-Finnish Europeans [28]). Its contribution to phenotypic manifestations compatible with FD and the controversy regarding its pathogenicity was repeatedly discussed in the literature [30–32]. The prevalence of the p.D313Y variant among our cohort (1.25%) is higher compared with the prevalence of the variant among patients with CNS manifestations (0.59%) [13]. This variant was also the most represented in our previous FD screening among unselected patients with stroke (0.91%) [13]. Given the results of both screenings and the recent finding that D313Y can induce ER stress [24], it might be hypothesised that this variant may play a role as a non-specific low-risk or modifying factor for some CNS diseases. This fact is underscored by the fact that ER stress has already been described in several CNS diseases, including MS [33].

One of our p.D313Y patients (patient 3.0.) showed minimal signs seen in patients with FD, requiring further follow-up by an ophthalmologist. Unfortunately, neither patient 4.0 nor her relatives agreed to a detailed examination at the FD centre. However, the neurological findings, imaging results, CSF analysis, and the clinical course of the disease do not support the influence of the p.D313Y allele on our patient’s clinical symptoms to date.

Therefore, our study brings a negative answer to the question of whether we should routinely screen all patients after the first clinical event suggestive of MS for FD. We did not demonstrate pathogenic carriage of the GLA variant in any of the 160 patients. However, the prevalence of VUS in our cohort was 2.50%, which we consider at least slightly higher compared with the healthy population based on available data [34]. Although the clinical significance of the detected GLA variants is still debated, they do not represent a standard indication for initiating FD-specific treatment. It should also be considered that the stress and psychosocial impact associated with repeated examinations while monitoring FD in VUS carriers with MS can negatively influence the MS course and can be harmful from this point of view [35].

However, given the potential overlap between clinical and laboratory features, it is still necessary to identify red flags and include FD in the differential diagnosis of MS. This is particularly the case when both diagnoses can explain clinical findings and MRI fails to differentiate between white matter hyperintensities of MS or FD origin. The absence of OCBs in CSF, especially in combination with previous findings, is also cautionary (Table 1). In such patients, it is advisable to complete an essential screening of organ manifestations, including urinalysis (proteinuria), eye examination, and cardiac examinations, including ECG and echocardiography. These investigations are commonly available and performed as part of the safety monitoring of patients with MS treated with some disease-modifying drugs. In the case of non-physiological findings during these examinations or organ manifestations associated with FD in the personal or family history, we recommend genetic testing.

Some limitations of the study should be noted. First, our population was relatively small. Second, further research is required in the VUS field concerning their clinical relevance and indication for treatment. Third, our analysis does not directly compare the prevalence of GLA variants in MS and healthy populations. Fourth, one patient with VUS did not agree to a detailed examination at the FD centre. And fifth, different screening methods were used for males and females. The GLA gene sequencing was performed only in males with abnormal AGAL activity and/or elevated lyso-GB3. In contrast, all samples obtained from female patients were sequenced. This may lead to underestimated frequency of several VUS or benign variants in hemizygous males in whom a high residual enzyme activity is preserved, and lyso-Gb3 remains low. Future studies investigating the whole spectrum of genetic variants should use GLA sequencing in all male patients. 

To sum up, the prevalence of FD in the population of suspected or definite MS patients seems to be very low. In general, the clinical significance of identified GLA gene variants is still debatable but is not a standard indication for FD therapy. Thus, our results do not support routine FD screening in all patients with possible MS. However, due to the frequent overlap of clinical and laboratory signs, there is still a need to look for red flags and include FD in the differential diagnosis of MS.

## Data Availability

Anonymised data not published within this article will be made available by request from any qualified investigator.
